# Axl and MerTK receptor tyrosine kinases maintain human macrophage efferocytic capacity in the presence of viral triggers

**DOI:** 10.1002/eji.201747283

**Published:** 2018-02-22

**Authors:** Aleksander M Grabiec, Anu Goenka, Mark E Fife, Toshifumi Fujimori, Tracy Hussell

**Affiliations:** ^1^ Department of Microbiology Faculty of Biochemistry Biophysics and Biotechnology Jagiellonian University Kraków Poland; ^2^ Manchester Collaborative Centre for Inflammation Research The University of Manchester Manchester UK

**Keywords:** Axl, IFN‐α, MerTK, Monocyte‐derived macrophages, TAM receptor

## Abstract

The requirement to remove apoptotic cells is equally important in homeostasis and inflammatory disease. In particular, during viral infections large quantities of infected cells undergo apoptosis and need to be efficiently cleared by phagocytes to prevent secondary necrosis. Although specific roles of several apoptotic cell sensors, such as the TAM (Tyro3, Axl, MerTK) receptor family, have been characterized in mouse models, little is known about their regulation and involvement in apoptotic cell uptake (efferocytosis) by human macrophages under inflammatory conditions. We show that whereas pro‐inflammatory stimuli consistently downregulated MerTK expression in human monocyte‐derived macrophages (MDMs), stimuli indicative of a viral infection, interferon‐α (IFN‐α) and the TLR3 ligand poly(I:C), specifically induced Axl expression and promoted binding of the bridging molecule Gas6. Axl induction by IFN‐α and poly(I:C) was associated with higher MDM efferocytic capacity compared to cells treated with other pro‐inflammatory stimuli, such as LPS and IFN‐γ. While MerTK blocking antibody uniformly suppressed apoptotic cell uptake by MDMs, Axl blocking antibody significantly reduced efferocytosis by poly(I:C)‐stimulated MDMs, but not by resting MDMs. Our observations demonstrate that Axl induction during viral infections contributes to maintaining macrophage capacity to engulf apoptotic cells, which may have important consequences for resolution of anti‐viral immune responses.

## Introduction

Macrophages regulate immune homeostasis through multiple mechanisms, among which the timely removal of apoptotic cells plays a crucial role. Phagocytosis of apoptotic cells, called efferocytosis, not only prevents secondary necrosis and the release of pro‐inflammatory necrotic cell debris, but also activates anti‐inflammatory signaling cascades in the phagocyte, thus facilitating resolution of inflammation and tissue repair 1, 2. Disruption of non‐inflammatory uptake of apoptotic cells by macrophages contributes significantly to the pathogenesis of several chronic inflammatory diseases, including systemic lupus erythematosus 3, asthma, chronic obstructive pulmonary disease and idiopathic pulmonary fibrosis 4.

In the last decade, great progress has been made in understanding the molecular mechanisms of efferocytosis and new classes of apoptotic cell recognition receptors have been identified. Phosphatidylserine (PtdSer) and other ‘eat‐me’ signals exposed on apoptotic cells are recognized by a plethora of receptors expressed by phagocytes, including proteins specifically responsible for apoptotic cell recognition, such as the TIM (T cell/transmembrane, immunoglobulin, and mucin) and TAM (Tyro3, Axl and MerTK) families 5, 6, as well as molecules involved in other processes, such as the scavenger receptor CD36, the LPS receptor CD14 and the angiogenesis regulator BAI1 (brain‐specific angiogenesis inhibitor‐1) 1. While some of them, such as TIM receptors, act only as tethering receptors, engagement of others has anti‐inflammatory signaling consequences 4. Apoptotic cell recognition by TAM receptors, through the bridging molecules Protein S or growth arrest specific 6 (Gas6), leads to receptor phosphorylation and the induction of suppressor of cytokine signaling‐1 (SOCS‐1) and SOCS‐3, which inhibit pro‐inflammatory signaling pathways triggered by cytokines and toll‐like receptor (TLR) ligands 6, 7. Mice lacking multiple TAM receptors develop systemic autoimmune disease 8 and important contributions of individual TAM family members to the resolution of inflammation have recently been demonstrated in mouse models 9, 10.

In mice, MerTK is expressed on all mature tissue macrophages, whereas Axl expression is restricted to specific macrophage populations: alveolar/airway macrophages, Kupffer cells in the liver and red pulp macrophages in the spleen 11, 12. Axl is also upregulated on murine macrophages by inflammatory stimuli 11, 12, suggesting a central role for Axl in apoptotic cell clearance during inflammation. Indeed, Axl‐deficient alveolar macrophages display reduced efferocytic capacity and we have shown enhanced morbidity to influenza virus infection in mice lacking Axl expression, associated with accumulation of dead cells in the lung 11. However, comparatively little is known about the role of Axl in apoptotic cell clearance by human macrophages. Studies of TAM receptors in monocyte‐derived macrophages (MDMs), which are the most accessible and commonly used model for primary human macrophages, have predominantly focused on MerTK, which is highly expressed on differentiated MDMs and regulated by pro‐ and anti‐inflammatory mediators 13, 14. The expression pattern of Axl and its involvement in efferocytosis by MDMs remain unknown. In this study, we show that specific induction of Axl on MDMs by inflammatory stimuli associated with viral infections contributes to macrophage capacity to engulf apoptotic cells.

## Results and discussion

### Viral triggers induce Axl expression on macrophages and promote Gas6 binding

**Figure 1:**
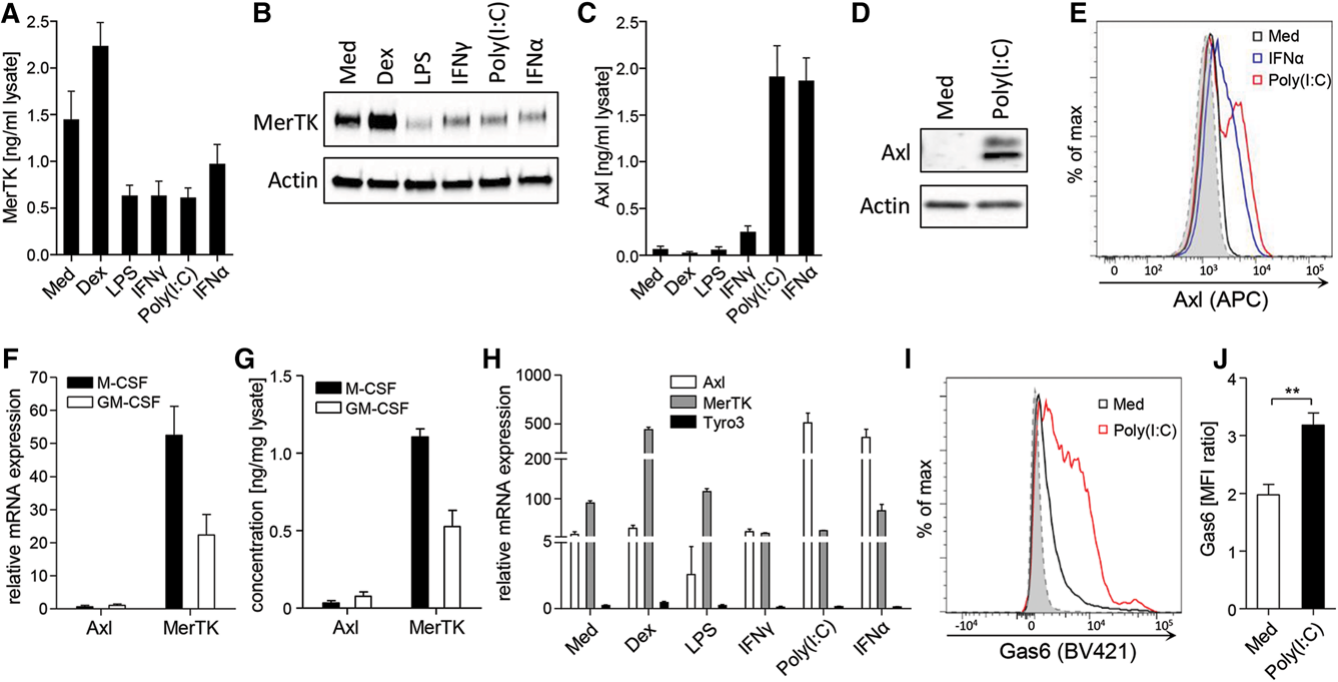
Inflammatory stimuli associated with viral infections upregulate Axl and promote Gas6 binding to macrophages. (A–E) MDMs were stimulated for 24 h with dexamethasone (Dex), LPS, IFN‐γ, poly(I:C) or IFN‐α. (A) MerTK and (C) Axl protein expression determined by ELISA of total cell lysates (mean+SEM, *n* = 4). (B) MerTK and (D) Axl protein expression analyzed by western blotting; actin was used as loading control. A representative of two to three independent experiments is shown. (E) Flow cytometric analysis of Axl expression on MDMs stimulated with poly(I:C) or IFN‐α for 24 h. Representative histograms of two independent experiments are shown; dotted line/shaded: FMO control. (F) Axl and MerTK relative mRNA and (G) protein expression in MDMs differentiated by 6‐day culture in M‐CSF or GM‐CSF determined by qPCR (mean+SEM, *n* = 6) and by ELISA of total cell lysates (mean+SEM, *n* = 3), respectively. (H) Axl, MerTK and Tyro3 relative mRNA expression in MDMs stimulated as in (A–C) analyzed by qPCR (mean+SEM, *n* = 2–3). (I and J) Flow cytometric analysis of Gas6 binding to MDMs stimulated with poly(I:C) for 24 h. (I) Representative of four independent experiments is shown; dotted line/shaded: isotype control. (J) Ratio of Gas6 to isotype control geometric mean fluorescence intensity (MFI)+SEM in unstimulated and poly(I:C)‐stimulated MDMs (*n* = 4). *p < 0.01, paired *t*‐test. ELISA and qPCR samples were assayed in duplicate.

We initiated this study by profiling changes in expression of the TAM receptors Axl and MerTK in M‐CSF‐differentiated MDMs stimulated with a range of pro‐ and anti‐inflammatory stimuli. In line with previous reports 13, 14, dexamethasone treatment caused upregulation of MerTK expression, whereas pro‐inflammatory signals caused its down‐regulation (Fig. 1A and B). AXL expression, on the other hand, was only up‐regulated by stimuli associated with viral infections: the TLR3 ligand poly(I:C) or interferon‐α (IFN‐α) (Fig. 1C–E and Supporting Information Fig. 1). The Axl and MerTK expression pattern was independent of the MDM differentiation method: GM‐CSF‐differentiated MDMs also expressed high levels of MerTK (albeit significantly lower compared to M‐CSF‐differentiated MDMs) and low levels of Axl (Fig. 1F–G), which were upregulated upon IFN‐α or poly(I:C) treatment (data not shown). Importantly, in all tested conditions MDMs expressed negligible levels of Tyro3 mRNA (Fig. 1H), indicating that Axl and MerTK are the sole TAM receptors involved in efferocytosis by human macrophages. The bridging ligand Gas6 is constitutively bound to Axl expressed on mouse and human alveolar macrophages 11, 15 and, consistently, poly(I:C) stimulation promoted increased binding of endogenously produced Gas6 to MDMs (Fig. 1I–J), which may prime these cells for apoptotic cell recognition via Axl. These observations indicate that upregulation of Axl may partly compensate for reduced expression of MerTK to maintain macrophage efferocytic capacity during viral infection.

The observation that induction of Axl by poly(I:C) and IFN‐α was associated with downregulation of MerTK also suggests that, unlike mouse airway macrophages, which express high levels of both Axl and MerTK 11, human macrophages preferentially express elevated levels of either Axl or MerTK (or low levels of both receptors) depending on the conditions or local microenvironment. Consistently, we have shown that human airway macrophages constitutively express high levels of Axl, but not MerTK 15. The functional consequences of this reciprocal regulation remain unclear, but are important to elucidate in future studies since Axl has higher affinity for the bridging ligand Gas6 compared to MerTK 6. While TAM receptor activation is generally associated with anti‐inflammatory responses 7, it is possible that subtle differences in transcriptional programs triggered by Axl and MerTK may exist and that Axl‐induced signaling specifically regulates resolution of viral infection‐induced inflammation.

### Inflammatory stimuli have differential effects on efferocytosis by human macrophages

**Figure 2:**
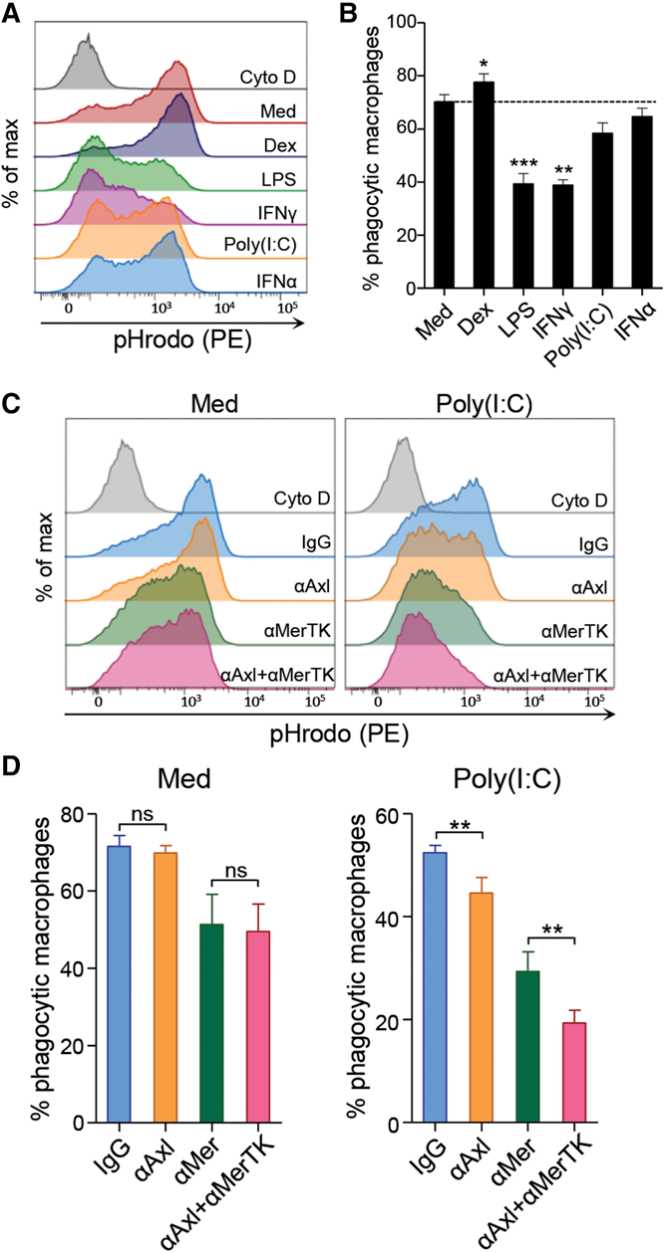
Axl contributes to efferocytosis by poly(I‐C)‐stimulated macrophages. (A and B) Uptake of pHrodo‐labeled apoptotic Jurkat cells by MDMs stimulated as in Fig. 1A analyzed by flow cytometry. MDMs treated with cytochalasin D (Cyto D) were used as negative control. (A) Representative histograms and (B) the mean percentage of phagocytic MDMs+SEM (*n* = 5) are shown. *p < 0.05, **p < 0.01, One‐way ANOVA followed by Bonferroni multiple comparison test. (C and D) Uptake of pHrodo‐labeled apoptotic Jurkat cells by MDMs that were left unstimulated or were stimulated for 24 h with poly(I:C) in the presence of control goat IgG, Axl blocking antibody, MerTK blocking antibody or a combination of both. (C) Representative histograms and (D) the mean percentage of phagocytic MDMs+SEM (*n* = 5) are shown. *p < 0.05, **p < 0.01, paired *t*‐test.

To gain more insight into the functional consequences of differential Axl and MerTK regulation we analyzed the efferocytic capacity of MDMs treated with pro‐ and anti‐inflammatory stimuli using pHrodo‐labelled apoptotic Jurkat cell uptake as a model. Untreated MDMs displayed high efferocytic capacity, which was further enhanced by dexamethasone (Fig. 2A and B). This is likely explained by increased MerTK expression after dexamethasone treatment 13, 14. In contrast, efferocytosis in the presence of LPS or IFN‐γ was significantly less efficient with only ∼40% of macrophages taking up apoptotic cells. Incubation of MDMs with IFN‐α or poly(I:C) resulted in a slight reduction of MDM efferocytic activity that was, however, less pronounced compared to LPS or IFN‐γ stimulation and did not reach statistical significance (Fig. 2A and B and Supporting Information Fig. 2). It is interesting that different stimuli affect MDM efferocytic capacity to varying degrees as it is believed that macrophage inflammatory stimulation globally reduces this function 16. However, influenza virus is reported to increase efferocytic activity of mouse alveolar macrophages during infection, which is dependent on soluble factors released by apoptotic influenza‐infected cells 17. Our results may thus reflect a specific process that sustains macrophage capacity to engulf virus‐infected cells undergoing apoptosis while mounting a rapid inflammatory response to viral infection. We hypothesize that Axl, which is specifically induced by IFN‐α and TLR3 ligation in MDMs, is involved in this process.

### Axl facilitates apoptotic cell uptake by human macrophages

To determine the relative contribution of Axl and MerTK to efferocytosis by MDMs, we utilized Axl and MerTK‐blocking antibodies which prevent TAM receptor‐mediated apoptotic cell uptake. MerTK blocking antibody partly prevented apoptotic cell uptake by both unstimulated and poly(I:C)‐stimulated MDMs, suggesting that even reduced levels of MerTK after poly(I:C) treatment significantly contribute to MDM efferocytosis. In contrast, Axl blocking antibody alone or in combination with anti‐MerTK antibody significantly reduced apoptotic cell engulfment by MDMs only after poly(I:C) stimulation, but not in unstimulated MDMs (Fig. 2C and D). These findings suggest that, although MerTK plays the most prominent role in apoptotic cell uptake by MDMs, selective induction of Axl by triggers associated with anti‐viral responses might contribute to the efferocytic capacity of macrophages during viral infection or at sites which are frequently exposed to viral challenges. It should be noted that efferocytosis was not completely blocked even after simultaneous treatment with Axl and MerTK blocking antibodies, suggesting the involvement of other receptors in this process.

Collectively, these observations imply that not only MerTK, but also Axl expression and activity on human macrophages play an important role during viral infections. Consistently, Axl‐expressing cells accumulate in the lungs of mice infected with influenza and Axl is required for resolution of pulmonary inflammation upon viral infection 11. We show that Axl is induced on MDMs by IFN‐α and, from our previous research, Axl expression on human alveolar macrophages is also likely dependent on IFN signaling 15. Axl is also induced by IFNs on murine dendritic cells 7 and is involved in the generation of anti‐viral T‐cell immunity and viral clearance 10, confirming an important role of the IFN‐Axl axis in the resolution of inflammation upon viral infection. In a recent study, Zagorska et al. demonstrated that MerTK is the main apoptotic cell recognition receptor in homeostasis, whereas Axl mediates efferocytosis during inflammation 12. Our data suggest that while MerTK is the main regulator of efferocytosis in human macrophages both under homeostatic and inflammatory conditions, induction of Axl is specifically involved in apoptotic cell clearance by cells exposed to viral triggers. It remains to be tested if a similar specificity of Axl function can be observed in human tissue macrophages during bacterial and viral infections, and whether compounds that upregulate Axl could facilitate phagocytosis of virus‐infected apoptotic cells. In that regard it is noteworthy that treatment with type I IFN, which maintains Axl expression on human airway macrophages 15, reduced disease exacerbations related to respiratory viral infection in patients with severe asthma 18. Airway macrophages from these patients are known to be defective in their efferocytic function 4.

Inhibition of Axl in the clinic is of high current interest due its oncogenic function in hematological and epithelial malignancies and a small‐molecule Axl inhibitor BGB324 is currently being evaluated in clinical trials 19. However, in non‐malignant conditions, such as acute inflammation following viral infection, inhibition of Axl may enhance inflammation by suppressing efficient elimination of apoptotic cells by macrophages 10, 11. Clinical trials of Axl inhibitors should therefore be designed with caution. Additional studies are also needed to delineate whether recognition of apoptotic cells by Axl and MerTK have different effects on the resulting anti‐inflammatory phenotype of macrophages, and characterize the roles of Axl expressed on human tissue macrophages, such as alveolar macrophages, during viral infections.

## Concluding remarks

Apoptotic cells that are not cleared undergo secondary necrosis that adds to the inflammatory environment. It is thus vital that a process exists whereby the additional apoptotic burden caused by inflammatory cell recruitment is dealt with. Our results indicate that this is facilitated during viral infection by an upregulation of the TAM receptor, Axl, which cooperates with the main apoptotic cell sensor in human macrophages, MerTK, in maintaining their efferocytic capacity. Many inflammatory diseases are prolonged by inefficient apoptotic cell clearance which could be tackled by administering therapeutics that up‐regulate Axl, such as type I interferons.

## Materials and methods

### Cell isolation and culture

Leukocyte cones with healthy donor peripheral blood were obtained from the National Blood Transfusion Service (Manchester, UK). Isolation of peripheral blood mononuclear cells (PBMCs) was performed by Ficoll‐Paque density gradient centrifugation. Human CD14 MicroBeads (Miltenyi Biotec) were then used to purify monocytes from PBMCs by magnetic‐activated cell sorting. Monocytes were plated at 5 × 10^5^ cells/mL in RPMI1640 medium containing 10% FCS, 100 U/mL penicillin, 100 μg/mL streptomycin, 2 mM L‐glutamine and 50 ng/mL M‐CSF or GM‐CSF (both from Peprotech), and cultured for 6 days. After differentiation into macrophages, culture medium was replaced with M‐CSF/GM‐CSF‐free medium and cells were stimulated with with 50 ng/mL IFN‐λ (Peprotech), 1000 U/mL IFN‐α (R&D Systems), 50 μg/mL poly(I:C), 100 ng/mL LPS (both from Invivogen) or 1 μM dexamethasone (Sigma‐Aldrich) for 24 h. Blood collection and processing was approved by the University of Manchester ethics committee.

### Total Axl/MerTK ELISA and western blotting

Macrophages were lysed in lysis buffer containing 1% Igepal CA‐630, 20 mM Tris pH 8.0, 137 mM NaCl, 10% glycerol, 2 mM EDTA and protease inhibitor cocktail (Sigma‐Aldrich). Axl and MerTK protein levels were analyzed in cell lysates using total (t)Axl and (t)MerTK, ELISA kits (R&D Systems) according to the manufacturer's instructions. Alternatively, protein expression was determined by western blotting as described before 15 using antibodies recognizing MerTK (D21F11) (Cell Signaling Technology), Axl (M20) or actin (I19‐R) (both from Santa Cruz Biotechnology). Membranes were visualized using a ChemiDoc MP Imaging System (BIO‐RAD).

### RNA extraction and quantitative PCR (qPCR)

RNA was extracted using RNeasy micro kit (QIAGEN) and equal amounts of RNA were reverse transcribed using High‐Capacity RNA‐to‐cDNA Kit (Life Technologies). qPCR reactions were performed on a QuantStudio 12K Flex PCR system using TaqMan Fast Universal PCR Master Mix and TaqMan expression assays (all from Life Technologies). QuantStudio 12K Flex Software (Life Technologies) was used for data analysis. mRNA expression of Axl, MerTK and Tyro3 was calculated relative to average expression of GAPDH and RPLP0.

### Flow cytometric analyses

Macrophages were detached and stained with Zombie UV Flexible Viability dye (BioLegend) for 20 min. Human TruStain FcX blocking solution (BioLegend) was then added for 10 min to block Fc receptors. Cells were washed with FACS buffer (PBS supplemented with 1% FCS and 2 mM EDTA), stained with APC‐conjugated anti‐Axl (FAB154A) and biotinylated anti‐Gas6 (BAF885) or control biotinylated goat IgG (BAF108) antibodies (all from R&D Systems) for 20 min at 4°C, and washed again. Macrophages were then incubated with BV421‐conjugated streptavidin (BioLegend) for 20 min at 4°C. After washing with FACS buffer cells were fixed by 10 min incubation with 2% paraformaldehyde solution in PBS and were acquired on a BD FACS Canto II. Results were analyzed using FlowJo software. Gating strategy is shown in Supporting Information Fig. 1.

### Apoptotic cell uptake assay

Jurkat T cells were treated with 1 μM camptothecin (Merck‐Millipore) for 4 h to induce apoptosis and labeled with 100 ng/mL pHrodo‐SE (Life Technologies) for 30 min at 37°C. MDMs were stimulated as described above or treated with control goat IgG (AB‐108‐C, 5 μg/mL), goat anti‐MerTK (AF891, 2.5 μg/mL) or goat anti‐Axl (AF154, 5 μg/mL) antibodies (all from R&D Systems) in the presence or absence of poly(I:C) for 24h prior to incubation with apoptotic cells (5:1 ratio of apoptotic cells:MDMs) for 60 min. Cells were detached and analyzed on a BD FACS Canto II (BD Biosciences). MDMs treated with 5 μg/mL cytochalasin D (Sigma‐Aldrich) were used as a negative control and percentages of phagocytic macrophages were calculated using FlowJo software (TreeStar). Gating strategy is shown in Supporting Information Fig. 2.

## Statistical analyses

Data are presented as the mean±SEM. Parametric tests (paired t‐tests or one‐way ANOVA) were used for comparisons between groups. *p* values <0.05 were considered statistically significant.

## Conflict of interest

T.H. is funded by GlaxoSmithKline and AstraZeneca. All other authors declare no commercial or financial conflict of interest.

**Abbreviations**
MDMs: Monocyte‐derived macrophages
TAM: Tyro3, Axl, MerTK

## Supporting information

Peer review correspondence

Supporting Information Figure S1
Supporting Information Figure S2
