# Supplementary material for: Axl and MerTK receptor tyrosine kinases maintain human macrophage efferocytic capacity in the presence of viral triggers
Source: Eur J Immunol. 2018 Feb 22;48(5):855–60. doi: 10.1002/eji.201747283 (PMC6001567; doi:10.1002/eji.201747283)
Supplement: Supplementary file 2 — Supporting Information Figure S1 Supporting Information Figure S2 [file EJI-48-855-s002.pdf]

European Journal of Immunology

Supporting Information

for

DOI 10.1002/eji.201747283

Aleksander M Grabiec, Anu Goenka, Mark E Fife, Toshifumi Fujimori and Tracy Hussell

Axl and MerTK receptor tyrosine kinases maintain human macrophage efferocytic capacity in the presence of viral triggers

© 2018 The Authors. *European Journal of Immunology* published by WILEY-VCH Verlag GmbH & Co. KGaA, Weinheim.

Supporting Information

Axl and MerTK receptor tyrosine kinases maintain human macrophage efferocytic capacity in the presence of viral triggers.

Aleksander M Grabiec, Anu Goenka, Mark E Fife, Toshifumi Fujimori and Tracy Hussell

Supplementary figures and figure legends

Figure S1

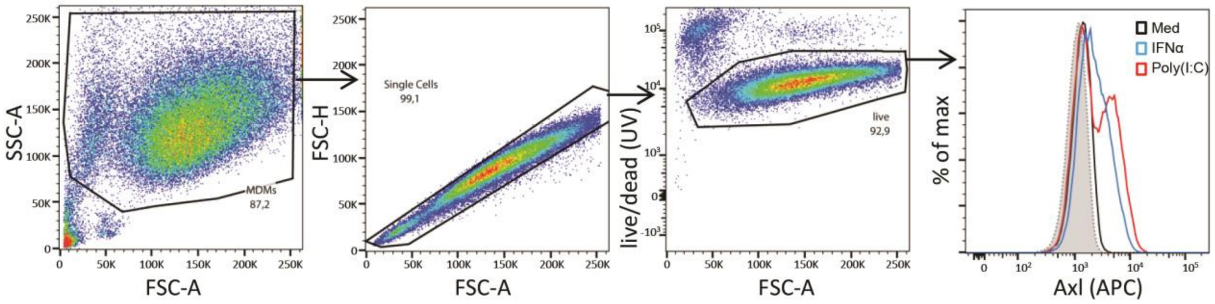

Figure S1. Gating strategy used in Axl flow cytometric analyses. Live single cells were selected based on FSC and SSC values and live/dead staining. The histogram of Axl surface expression is shown. Dotted line/shaded: FMO control. Because MDMs are an isolated cell population, lineage staining was not included. The same gating strategy was used in flow cytometric analyses of Gas6 binding to MDMs. FSC-A, forward scatter-area; FSC-H, forward scatter-height; SSC-A, side scatter-area.

Figure S2

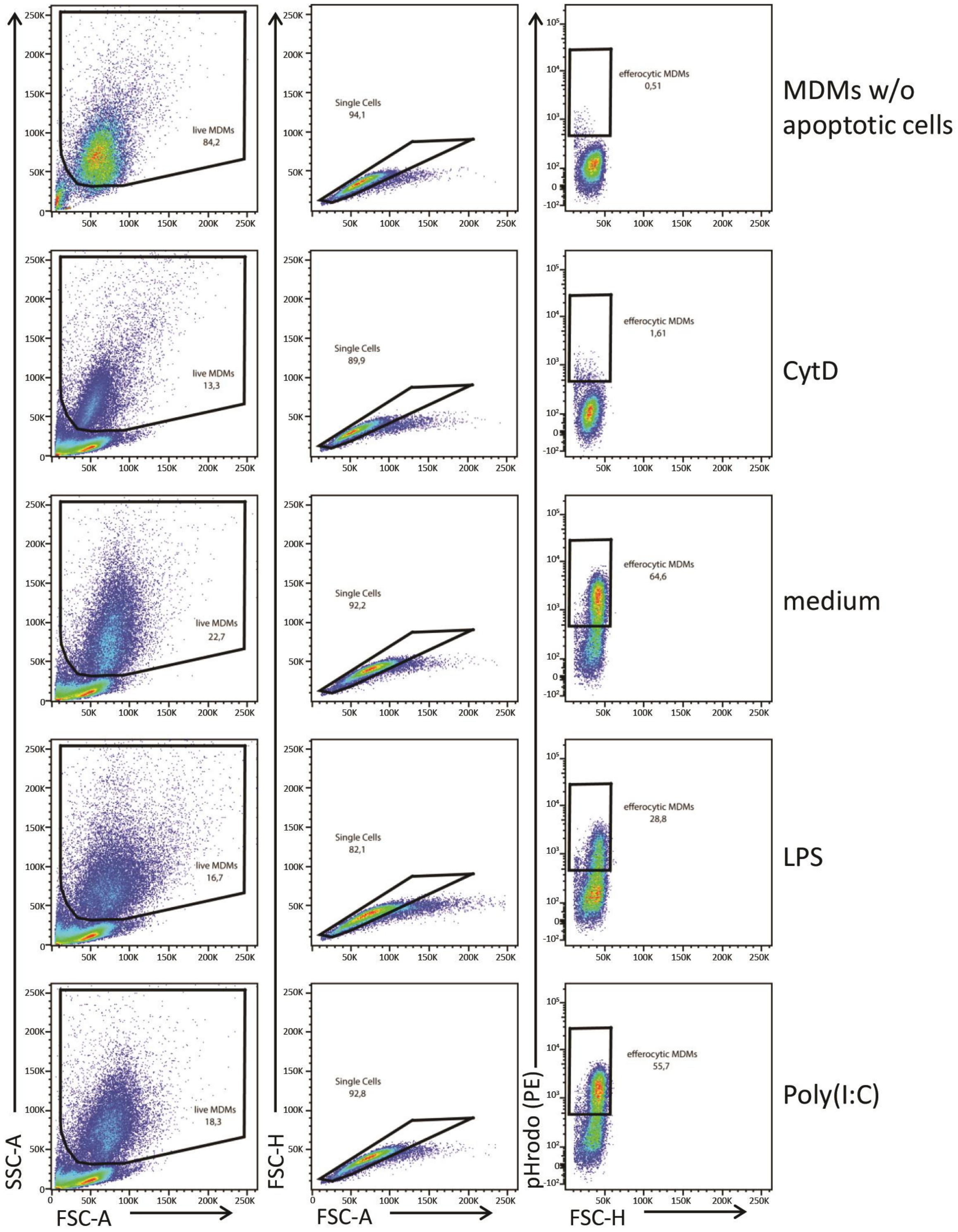

Figure S2. Gating strategy used in apoptotic cell uptake assays. Live MDMs were separated from apoptotic Jurkat cells based on FSC and SSC values (left panels – MDMs were defined as $FSC^{hi}SSC^{hi}$). Single cells were gated (middle panels) and the numbers of pHrodo-positive phagocytic MDMs were determined (right panels).

Cytochalasin D (Cyt D)-treated cells were used as a negative control to distinguish MDMs that did not take up apoptotic cells. Representative dot plots for selected conditions of 5 independent experiments are shown. FSC-A, forward scatter-area; FSC-H, forward scatter-height; SSC-A, side scatter-area.
