# Supplementary material for: Axl and MerTK receptor tyrosine kinases maintain human macrophage efferocytic capacity in the presence of viral triggers
Source: Eur J Immunol. 2018 Feb 22;48(5):855–60. doi: 10.1002/eji.201747283 (PMC6001567; doi:10.1002/eji.201747283)
Supplement: Supplementary file 1 — Peer review correspondence [file EJI-48-855-s001.pdf]

European Journal of Immunology

Axl and MerTK receptor tyrosine kinases maintain human macrophage efferocytic capacity in the presence of viral triggers.

Aleksander M. Grabiec, Anu Goenka, Mark E Fife, Toshifumi Fujimori and Tracy Hussell

Correspondence: Dr. Aleksander M. Grabiec, Department of Microbiology, Faculty of Biochemistry, Biophysics and Biotechnology, Jagiellonian University, Gronostajowa 7, 30-387 Kraków, Poland.

Review Timeline:	Submission date:	12-Aug-2017
	First Editorial decision:	29-Aug-2017
	Revision received:	13-Jan-2018
	Accepted:	30-Jan-2018

Handling Executive Committee member: Prof. Kenneth Murphy

Please note that the correspondence below does not include the standard editorial instructions regarding preparation and submission of revised manuscripts, only the scientific revisions requested and addressed.

First Editorial Decision

29-Aug-2017

Dear Dr. Grabiec,

Manuscript ID eji.201747283 entitled "The Axl receptor tyrosine kinase maintains macrophage efferocytic capacity in the presence of viral triggers." which you submitted to the European Journal of Immunology has been reviewed. The comments of the referees are included at the bottom of this letter.

Both reviewers expressed a positive opinion on your manuscript, Referee #2 in particular.
Referee #1 suggested some additional investigation, to further support the conclusions of the study.

A revised version of your manuscript that takes into account the comments of the referees will be reconsidered for publication. Should you disagree with any of the referees concerns, you should address this in your point-by-point response and provide solid scientific reasons for why you will not make the

requested changes.

You should also pay close attention to the editorial comments included below. **In particular, please edit your figure legends to follow Journal standards as outlined in the editorial comments. Failure to do this will result in delays in the re-review process.**

Please note that submitting a revision of your manuscript does not guarantee eventual acceptance, and that your revision will be re-reviewed by the referees before a decision is rendered.

If the revision of the paper is expected to take more than three months, please inform the editorial office. Revisions taking longer than six months may be assessed by new referees to ensure the relevance and timeliness of the data.

Once again, thank you for submitting your manuscript to European Journal of Immunology and we look forward to receiving your revision.

Yours sincerely,

Marta Vuerich

On behalf of
Prof. Kenneth Murphy

Dr. Marta Vuerich
Editorial Office
European Journal of Immunology
e-mail: ejied@wiley.com
www.eji-journal.eu

****

Reviewer: 1

Comments to the Author
The authors of the manuscript attempt to show that human MDMs maintain efferocytosis through Axl during viral inflammation. The results are novel in that they bridge unknowns between the mouse and

human and will further advance our understanding of the TAM receptors during inflammation in humans. However, the data does not necessarily support the authors' conclusions. Nevertheless, the lead author is a leader in the field of efferocytosis and her experience in the field shows the strength of the manuscript.

Comments/expts. that should be added to the publication:

(1) In Figure 1, a western blot of expression of MerTK on the various treatments is shown but not Axl. Because the manuscript centers on Axl's role in efferocytosis during inflammation mimicking viral infection, it would strengthen the paper to show that the protein level increases. The ELISA for MerTK is shown, which corroborates the MerTK western and the reciprocal western for Axl would strengthen the Axl ELISA.

(2) In Figure 2, the gating for positive apoptotic cell uptake is not presented. The reader should be able to clearly delineate what the authors are calling positive uptake of pHrodo labelled cells. The authors show in the graph that poly I:C does not have an effect on efferocytosis, but the flow cytometry does not indicate such results. Poly I:C seems to show a reduction in efferocytosis compared to media alone (Figure 2A). The authors should show their positive pHrodo gate in order to strengthen their argument that poly I:C treatment does not inhibit efferocytosis.

(3) Along those lines, the authors need to explain why blocking MerTK inhibits efferocytosis to a greater extent when compared to Axl when MDMs are treated with poly I:C (figure 2 C & D right panels). If Axl is indeed maintaining efferocytosis during viral infection, why then, if MerTK expression is reduced and Axl is expressed, does MerTK still predominate in efferocytic clearance during poly I:C stimulation? This point is further enhanced by the finding that overall efferocytic potential is reduced when polyI:C is administered (see previous statement and compare % phagocytic macrophages treated with media or poly I:C in figure 2D). This finding points to the reduction of MerTK as the culprit and that Axl is not as prominent as the authors suggest. It is possible that the Axl blocking antibody used is a poor blocking antibody or not used in high enough concentrations. In that case, knocking down expression of Axl should truly inhibit efferocytosis during viral inflammation. Addressing this point and conducting this experiment is critical to support the author's hypothesis.

(4) The authors did not show any expression of Tyro. As Tyro is the third member of the TAM receptors, it is necessary to show its expression during the various treatments. It is possible that Tyro expression is increased/decreased during the treatments as well.

In conclusion, the authors would be better suited stating that during viral inflammation MerTK is still the dominant efferocytic receptor and that Axl plays a minor role in regulating efferocytosis in human MDM.

This does not preclude that there may be an effect in the human lung macrophage population during viral infection. However, the data does not corroborate their conclusions that Axl maintains the efferocytoic capacity of human macrophages.

Reviewer: 2

Comments to the Author
In this paper Grabiec et al. investigate apoptotic cell recognition receptors, specifically Axl and MerTK on primary human monocyte-derived macrophages. They find that inflammatory triggers indicative of a viral infection promote expression of Axl and suppress expression of MerTK. In contrast, inflammatory triggers associated to bacterial pathogens do not induce Axl, but also suppress MerTK. While macrophages stimulated with signals of viral infection maintain effectorcytosis, macrophages stimulated with signals of bacterial infection show reduced effectorcytosis. Given that effectorcytosis in macrophages activated with viral-infection-associated triggers partially depends on Axl, the authors conclude that Axl mediates sustained effectorcytosis, which is critical to clear apoptotic cells, during viral infections.
The paper is overall interesting, well written and the data are well presented. The experiments are technically sound and the conclusions drawn are supported by the data. A limitation of the paper is that the authors do not show physiological relevance of their findings, e.g. in cells of patients with viral infections compared to healthy controls/patients suffering from bacterial infections. However, given that Axl inhibitors are being evaluated in clinical cancer trials, their findings are of clinical interest and warrant further investigation. In sum, I recommend the paper for publication as short communication in the EJI.

First Revision – authors' response

18-Sep-2017

We would like to thank the reviewers for their helpful commentary which has allowed us to improve the quality of our manuscript. A point by point response to each reviewer follows:

Reviewer: 1

(1) In Figure 1, a western blot of expression of MerTK on the various treatments is shown but not Axl. Because the manuscript centers on Axl's role in efferocytosis during inflammation mimicking viral infection, it would strengthen the paper to show that the protein level increases. The ELISA for MerTK is shown, which corroborates the MerTK western and the reciprocal western for Axl would strengthen the Axl ELISA.

Response: In the revised manuscript we have included a western blot showing upregulation of Axl by the TLR3 ligand poly(I:C) in M-CSF-differentiated MDMs (revised Figure 1E). Induction of the Axl protein by viral triggers (poly(I:C) and/or IFNα) is now confirmed using three independent methods: cell lysate ELISA, flow cytometry and western blotting. Importantly, all three methods also show that unstimulated MDMs express negligible or undetectable levels of the Axl protein.

(2) In Figure 2, the gating for positive apoptotic cell uptake is not presented. The reader should be able to clearly delineate what the authors are calling positive uptake of pHrodo labelled cells. The authors show in the graph that poly I:C does not have an effect on efferocytosis, but the flow cytometry does not indicate such results. Poly I:C seems to show a reduction in efferocytosis compared to media alone (Figure 2A). The authors should show their positive pHrodo gate in order to strengthen their argument that poly I:C treatment does not inhibit efferocytosis.

We thank the reviewer for pointing out this inconsistency in our manuscript. First, we confirm that indeed poly(I:C) does have a suppressive effect on MDM efferocytosis, though it is much less pronounced and consistent compared to suppression of apoptotic cell uptake by LPS or IFNγ (based on that we postulate the existence of a compensatory mechanism, namely Axl induction, which is responsible for this intermediate efferocytic phenotype despite downregulation of MerTK). We have rephrased all sections of the text and explained that this is a quantitative rather than qualitative difference in suppression of efferocytosis by inflammatory stimuli. Second, we have included Supporting Information Figure S2, which shows the gating strategy that was used in uptake assays. We hope this clarifies how macrophages that engulfed apoptotic cells are defined in our study.

(3) Along those lines, the authors need to explain why blocking MerTK inhibits efferocytosis to a greater extent when compared to Axl when MDMs are treated with poly I:C (figure 2 C & D right panels). If Axl is indeed maintaining efferocytosis during viral infection, why then, if MerTK expression is reduced and Axl is expressed, does MerTK still predominate in efferocytic clearance during poly I:C stimulation? This point is further enhanced by the finding that overall efferocytic potential is reduced when polyI:C is administered (see previous statement and compare % phagocytic macrophages treated with media or poly I:C in figure 2D). This finding points to the reduction of MerTK as the culprit and that Axl is not as prominent as the authors

suggest. It is possible that the Axl blocking antibody used is a poor blocking antibody or not used in high enough concentrations. In that case, knocking down expression of Axl should truly inhibit efferocytosis during viral inflammation. Addressing this point and conducting this experiment is critical to support the author's hypothesis.

Response: We agree with the reviewer that independent verification of the roles of Axl and MerTK in efferocytosis would greatly increase the strength of our study. We have therefore attempted to knock down Axl or MerTK using siRNA analyzed the effects of gene silencing on MDM efferocytosis. Unfortunately, neither MerTK, nor Axl gene silencing affected apoptotic cell uptake by MDMs regardless of the presence or absence of inflammatory stimuli (figure for reviewer below, panel A). This negative result can be possibly explained by a relatively poor knockdown efficiency: both Axl and MerTK siRNA transfection resulted in approximately 40-50% reduction of target gene mRNA expression (figure for reviewer below, panel B). This is likely insufficient to translate into significant reduction of protein expression levels. Alternatively, silencing of TAM receptors might cause an induction of other apoptotic cell recognition receptors as a compensatory mechanism, which in turn restores MDM efferocytic capacity. It is important to note that primary MDMs are well known to be difficult to transfect and efficient delivery of siRNA without robust cell activation is challenging. Validation of our results by gene manipulation would therefore require an alternative approach, such as lentiviral delivery of shRNA constructs or CRISPR/Cas technology.

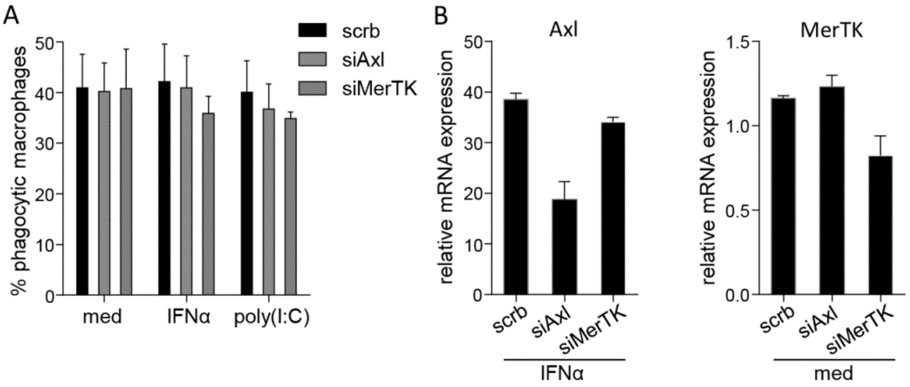

In light of the observation that MerTK blocking antibody suppresses efferocytosis both in the absence and presence of poly(I:C), we have rephrased these parts of the text and emphasized that even reduced expression of MerTK plays a key role in apoptotic cell uptake in MDMs. In contrast, Axl is only involved in efferocytosis after treatment with viral stimuli.

(4) The authors did not show any expression of Tyro. As Tyro is the third member of the TAM receptors, it is necessary to show its expression during the various treatments. It is possible that Tyro expression is increased/decreased during the treatments as well.

Response: Following the reviewer’s suggestion, we have analyzed mRNA expression profiles of Tyro3, Axl and MerTK in MDMs treated with pro- and anti-inflammatory stimuli. MDMs expressed negligible levels of Tyro3 mRNA compared to MerTK and Axl and no significant up- or down-regulation was observed in any tested experimental condition (revised Figure 1H). In line with the protein data, Axl mRNA was strongly induced by IFNα and poly(I:C)

treatment, whereas MerTK was upregulated by dexamethasone. Furthermore, in a separate analysis we found that human monocytes, MDMs and airway macrophages express similarly low levels of Tyro3 mRNA, suggesting that Axl and MerTK are the only TAM receptors involved in efferocytosis by human monocytes/macrophages (see figure for reviewer below).

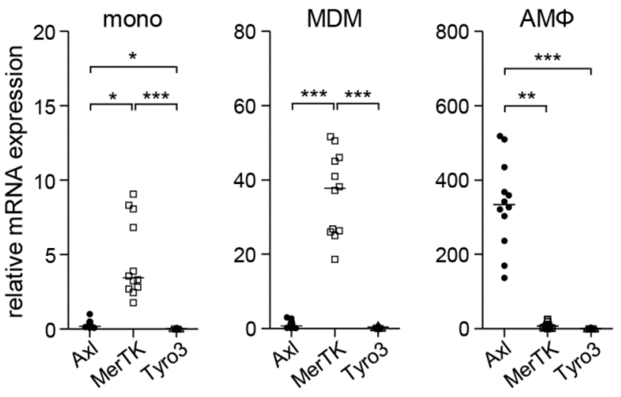

In conclusion, the authors would be better suited stating that during viral inflammation MerTK is still the dominant efferocytic receptor and that Axl plays a minor role in regulating efferocytosis in human MDM. This does not preclude that there may be an effect in the human lung macrophage population during viral infection. However, the data does not corroborate their conclusions that Axl maintains the efferocytoic capacity of human macrophages.

We agree with the reviewer that in the first version of our manuscript we overstated the role of Axl in MDM efferocytosis. We have modified the title, abstract and parts of the main text to emphasize that MerTK plays a key role in apoptotic cell uptake by MDMs both in the absence and presence of inflammatory stimuli. However, we believe that our results support the conclusion that induction of Axl is specifically involved in apoptotic cell engulfment by MDMs treated with stimuli associated with viral infections. Under this condition, both TAM receptors partly maintain MDM efferocytic capacity of MDMs and Axl plays a significant, albeit minor, role in this process.

Reviewer: 2

The paper is overall interesting, well written and the data are well presented. The experiments are technically sound and the conclusions drawn are supported by the data. A limitation of the paper is that the authors do not show physiological relevance of their findings, e.g. in cells of patients with viral infections compared to healthy controls/patients suffering from bacterial infections. However, given that Axl inhibitors are being evaluated in clinical cancer trials, their findings are of clinical interest and warrant further investigation. In sum, I recommend the paper for publication as short communication in the EJI.

Response: We thank the reviewer for his kind comments. We agree that analyses of cells isolated from patients with viral infections would strongly increase the impact of our work. In

our previous study, we demonstrated that alterations in Axl expression on airway macrophages contribute to the pathogenesis of severe asthma (Grabiec et al., JACI 2017). Airway macrophages from patients suffering from influenza infection or other respiratory viral infections would be required to validate the findings presented in this manuscript. Since such patients are difficult to recruit, in our future work we are planning to assemble a cohort of patients with asthma suffering from viral infection-related exacerbation. Analysis of airway macrophages from these patients compared to controls without exacerbations would provide a definitive answer regarding the role of Axl in pathophysiological conditions. However, this analysis is beyond the scope of this article and will be subject of our future studies.

European Journal of Immunology

Second Editorial Decision

17-Jan-2018

Dear Dr. Grabiec,

It is a pleasure to provisionally accept your manuscript entitled "Axl and MerTK receptor tyrosine kinases maintain macrophage efferocytic capacity in the presence of viral triggers." for publication in the European Journal of Immunology. For final acceptance, please follow the instructions below and return the requested items as soon as possible as we cannot process your manuscript further until all items listed below are dealt with.

Please note that EJI articles are now published online a few days after final acceptance (see Accepted Articles: http://onlinelibrary.wiley.com/journal/10.1002/(ISSN)1521-4141/accepted). The files used for the Accepted Articles are the final files and information supplied by you in Manuscript Central. You should therefore check that all the information (including author names) is correct as changes will NOT be permitted until the proofs stage.

We look forward to hearing from you and thank you for submitting your manuscript to the European Journal of Immunology.

Yours sincerely,
Eloho Etemire

on behalf of
Prof. Kenneth Murphy

Dr. Eloho Etemire
Editorial Office
European Journal of Immunology
e-mail: ejied@wiley.com
www.eji-journal.eu
